# Insecticide susceptibility in a planthopper pest increases following inoculation with cultured *Arsenophonus*

**DOI:** 10.1093/ismejo/wrae194

**Published:** 2024-10-07

**Authors:** Tingwei Cai, Pol Nadal-Jimenez, Yuanyuan Gao, Hiroshi Arai, Chengyue Li, Chunyan Su, Kayla C King, Shun He, Jianhong Li, Gregory D D Hurst, Hu Wan

**Affiliations:** State Key Laboratory of Agricultural Microbiology, Huazhong Agricultural University, Wuhan 430070, China; Hubei Hongshan Laboratory, Wuhan 430070, China; Department of Microbiology & Immunology, University of British Columbia, Vancouver, British Columbia V6T 1Z3, Canada; Hubei Insect Resources Utilization and Sustainable Pest Management Key Laboratory, College of Plant Science and Technology, Huazhong Agricultural University, Wuhan 430070, China; Department of Vector Biology, Liverpool School of Tropical Medicine, Liverpool L3 5QA, United Kingdom; Institute of Infection, Veterinary and Ecological Sciences, University of Liverpool, Liverpool L69 7ZB, United Kingdom; State Key Laboratory of Agricultural Microbiology, Huazhong Agricultural University, Wuhan 430070, China; Hubei Hongshan Laboratory, Wuhan 430070, China; Hubei Insect Resources Utilization and Sustainable Pest Management Key Laboratory, College of Plant Science and Technology, Huazhong Agricultural University, Wuhan 430070, China; Institute of Infection, Veterinary and Ecological Sciences, University of Liverpool, Liverpool L69 7ZB, United Kingdom; State Key Laboratory of Agricultural Microbiology, Huazhong Agricultural University, Wuhan 430070, China; Hubei Hongshan Laboratory, Wuhan 430070, China; Hubei Insect Resources Utilization and Sustainable Pest Management Key Laboratory, College of Plant Science and Technology, Huazhong Agricultural University, Wuhan 430070, China; State Key Laboratory of Agricultural Microbiology, Huazhong Agricultural University, Wuhan 430070, China; Hubei Hongshan Laboratory, Wuhan 430070, China; Hubei Insect Resources Utilization and Sustainable Pest Management Key Laboratory, College of Plant Science and Technology, Huazhong Agricultural University, Wuhan 430070, China; Department of Microbiology & Immunology, University of British Columbia, Vancouver, British Columbia V6T 1Z3, Canada; Hubei Insect Resources Utilization and Sustainable Pest Management Key Laboratory, College of Plant Science and Technology, Huazhong Agricultural University, Wuhan 430070, China; Hubei Insect Resources Utilization and Sustainable Pest Management Key Laboratory, College of Plant Science and Technology, Huazhong Agricultural University, Wuhan 430070, China; Institute of Infection, Veterinary and Ecological Sciences, University of Liverpool, Liverpool L69 7ZB, United Kingdom; State Key Laboratory of Agricultural Microbiology, Huazhong Agricultural University, Wuhan 430070, China; Hubei Hongshan Laboratory, Wuhan 430070, China; Hubei Insect Resources Utilization and Sustainable Pest Management Key Laboratory, College of Plant Science and Technology, Huazhong Agricultural University, Wuhan 430070, China

**Keywords:** symbiosis, insecticide susceptibility, plant pests, microbiology, entomology

## Abstract

Facultative vertically transmitted symbionts are a common feature of insects that determine many aspects of their hosts’ phenotype. Our capacity to understand and exploit these symbioses is commonly compromised by the microbes unculturability and consequent lack of genetic tools, an impediment of particular significance for symbioses of pest and vector species. Previous work had established that insecticide susceptibility of the economically important pest of rice, the brown planthopper *Nilaparvata lugens*, was higher in field-collected lineages that carry Ca. *Arsenophonus nilaparvatae.* We established Ca. *A. nilaparvatae* into cell-free culture and used this to establish the complete closed genome of the symbiont. We transformed the strain to express GFP and reintroduced it to *N. lugens* to track infection *in vivo*. The symbiont established vertical transmission, generating a discrete infection focus towards the posterior pole of each *N. lugens* oocyte. This infection focus was retained in early embryogenesis before transition to a diffuse somatic infection in late *N. lugens* embryos and nymphs. We additionally generated somatic infection in novel host species, but these did not establish vertical transmission. Transinfected planthopper lines acquired the insecticide sensitivity trait, with associated downregulation of the P450 xenobiotic detoxification system of the host. Our results causally establish the role of the symbiont in increasing host insecticide sensitivity with implications for insecticide use and stewardship. Furthermore, the culturability and transformation of this intracellular symbiont, combined with its ease of reintroduction to planthopper hosts, enables novel approaches both for research into symbiosis and into control of insect pest species.

## Introduction

Arthropods, perhaps more than any other terrestrial animal group, live in a microbial world [1]. They form symbioses with microorganisms within their gut and endosymbionts that live within their body [2]. Endosymbionts are commonly transmitted vertically, from female host to progeny, which has selected for reduced virulence of the microbe alongside a positive contribution to a broad variety of host traits [3]. These contributions include (but are not limited to) desiccation tolerance, anabolic capacity, defence against natural enemies, and vector competence [4–6]. As such, heritable symbionts have been suggested to be an “extended genome” of their host [2]. The traits they encode have major impacts on the ecology and evolution of arthropods, and additionally enable novel approaches for pest and vector control [7, 8]. For instance, symbiont impacts on the sensitivity of pest species to insecticide provide a pathway to exploitation—if the symbiont that increases pesticide sensitivity can be driven to high prevalence, it can allow reduced and more effective use of chemical insecticides with both economic and biodiversity benefits.

The unculturable nature of many symbionts represents a key impediment to understanding and exploiting these systems. Microbes that transmit vertically commonly have degraded genomes that prevent cell-free culture, preventing deployment of the tools of molecular microbiology that generally underpin functional analysis [9]. In many cases, our understanding of the phenotypic impacts of symbionts derives from association rather than causal experiments. Direct functional analysis has been achieved through alternative approaches, for instance directed experimental evolution and ectopic expression of target genes in host insects [10, 11]. However, these approaches are laborious and commonly require model insect species.

Recent research has extended the range of heritable symbionts that have been cultured *in vitro* [12]. For many years, *Arsenophonus nasoniae* was the sole vertically transmitted microbe growing in *in vitro* culture, likely facilitated by its mixed mode of transmission (horizontal and vertical) and large genome size [13]. Currently, the most sophisticated toolkit available is for *Sodalis*, an insect symbiont that has been cultured and transformed for onward study [14]. This system has recently been developed through a synthetic biology approach, adding function to *Sodalis* to enable development of a host dependent on external inputs of tyrosine and phenylalanine [15]. More recently, strains of *Serratia symbiotica* and Ca. Fukatsuia symbiont of aphids were cultured [16, 17]. The former was genetically manipulated and colonized the gut of the aphid host, but did not establish vertical transmission in the aphid, unlike the latter. These developments are important as they allow direct causal inference of symbiont phenotype and enable analysis of gene function through knock-out mutations. They also provide a basis for engineering gain-of-function into symbionts for testing hypotheses and potential application.

Our study focussed on *Arsenophonus* in the agriculturally important rice pest, the brown planthopper *N. lugens*. This planthopper produces economic losses both directly through feeding activity and indirectly through vectoring virus to the rice plant [18]. Ca. *A. nilaparvatae*, is a member of the third clade of *Arsenophonus*, termed the triatominarum clade, that comprises vertically transmitted symbionts of pest and vector hemipteran bugs such as aphids and triatomines [19–21]. The symbiosis in brown planthoppers is of particular interest for three reasons. First, Ca. *A. nilaparvatae* presence is associated with increased susceptibility of *N. lugens* to insecticides [22]. Individuals and lines that carry S type *Arsenophonus* died at lower insecticide dose. This sensitivity phenotype provides a pathway for exploitation of the symbiosis through enabling reductions in the use of chemical insecticide with both economic and biodiversity benefits. However, the linkage of microbe to the trait is currently through association and exclusion of other microbes, rather than testing through defined experimental introduction of a monoisolate. Second, understanding the insecticide-susceptibility trait in mechanistic terms may enable exploitation more widely in pest control in other symbioses. Finally, transformation of the symbiont and gain-of-function engineering may enable the development of strains expressing traits of importance—for instance, making the host refractory to virus propagation in the crop context.

We first established culture methods for this microbe and used this to complete the closed genome of the species. We then transformed the microbe to express GFP to enable us to track infection *in vivo*. We reintroduced this strain to *N. lugens* to establish patterns of vertical transmission and somatic propagation of the symbiont in its native host. We further investigated if the strain could establish somatically and vertically transmit in other host species, including the model organism *Drosophila melanogaster*. Finally, we formally tested the role of Ca. *A. nilaparvatae* in driving susceptibility of *N. lugens* to insecticides through comparing the phenotype of planthopper lines that had Ca. *A. nilaparvatae* established through transinfection of the isolated microbe compared to untreated controls.

## Materials and methods

### Insect material

An *Arsenophonus*-infected field population of *N. lugens* was originally collected from a rice paddy field in Xinyang City (31.58°N, 115.24°E), China, in 2017. *N. lugens* are maintained on rice seedlings at 28°C under 70%–80% relative humidity and a 16-hour light/8-hour dark photoperiod [23]. *Arsenophonus*-uninfected *N. lugens* (Ars^−^) in this population was allowed to expand. *S. furcifera* originally derived from rice paddy fields in Jingzhou City (30.34°N, 112.50°E), China, in 2023 and was maintained on the same conditions as *N. lugens. D. melanogaster* (strain Canton-S, CS) is maintained in the lab of the authors in a cornmeal-yeast agar medium [24]. Axenic flies were derived from dechorionated eggs as previously described [25]. This part is described in more detail in Supplementary Methods.

### Symbiont isolation, morphology *in vitro*, and identification through 16S rRNA gene sequence

Eggs of *N. lugens* were peeled off rice seedlings and surface sterilized by washing with 75% ethanol three times, before homogenization with sterile deionized water. The resultant liquid was spread without dilution on DNase agar (Qingdao Hope Bio-Technology Co. Ltd.). The medium was cultivated at 28°C under standard aerobic conditions. Subsequently, small bacterial colonies became evident after six days of growth. This bacterial strain underwent five generations of purification before identification and the 16S rRNA gene sequence of colonies was obtained from the PCR product amplified using primers 27F/1492R (Supplementary Table S1). The PCR protocol is described in more detail in Supplementary Methods.

### Symbiont genome sequencing, assembly, and annotation

Ca. *A. nilaparvatae* was grown in BHI media for five days. After this period, the culture was spun down in 50-ml centrifuge tubes at 20 000 g for 10 min. The collected pellet of Ca. *A. nilaparvatae* was rapidly transferred to liquid nitrogen. The Ca. *A. nilaparvatae* strain HZAU001 genomic DNA was extracted and sequenced using Nanopore and DNBSEQ platform at the Beijing Genomics Institute (BGI, Shenzhen, China). The cleaned Nanopore reads were assembled using Flye 2.9.3 [26] with the nanoraw mode. The assembled two closed circular contigs were polished with the DNBSEQ data using Pilon.1.24 [27] for six times until no changes were recorded. The resulting polished genome was annotated using the NCBI Prokaryotic Genome Annotation Pipeline (PGAP). Gene prediction was also performed on the Ca. *A. nilaparvatae* genome assembly by glimmer V3.02 with Hidden Markov models and multiple databases used to annotate gene functions [28]. Genome sequencing and analysis methods are described in more detail in Supplementary Methods.

### Genetic manipulation of *Ca A. nilaparvatae*

Fluorescent strains of Ca. *A. nilaparvatae* were obtained following the method as previously described [29]. A frozen strain of Ca. *A. nilaparvatae* was passaged over five generations, competent Ca. *A. nilaparvatae* cells established and mixed with plasmid pOM1-*gfp*. The mixture was then placed on ice and electroporated in a micropulser electroporator (Bio-Rad, UK). The transformed culture was allowed to recover before being plated on BHI agar containing spectinomycin (Sigma). After six days of incubation, colonies were checked for GFP fluorescence using an Olympus SZX16 stereoscope (Olympus, Japan). A high copy number of this plasmid (c. 320 copies/main chromosome) ensures faithful maintenance without segregation. The genetic manipulation protocol is described in more detail in Supplementary Methods.

### Artificial infection of *N. lugens* with Ca. *A. nilaparvatae*

Two main methods were used for artificial infection: feeding and injection. In brief, a bacterial suspension of Ca. *A. nilaparvatae*-GFP was prepared. In the feeding group, 20 nymphs of *N. lugens* were released into a glass tube (2 × 15 cm) containing 100 μl of bacterial suspension, sandwiched between two membranes of stretched parafilm [30]. Samples were collected after 24 h feeding and remaining *N. lugens* nymphs transferred to normal rice seedlings for incubation until the next generation was sampled. In the injection treatment, each *N. lugens* nymph was injected with 50 nl of bacterial suspension into the ventral abdomen and then placed on rice seedlings for rearing. Sample collection time points for the injection treatment were the same as those for the feeding treatment.

### Specific detection and relative quantification of Ca. *A. nilaparvatae*

Total genomic DNA was extracted from the whole bodies of *N. lugens*. We employed three pairs of primers (ArsftsK, Ars16S, and Arsfrag, Supplementary Table S1) for the specific detection of infection of Ca. *A. nilaparvatae.* The relative Ca. *A. nilaparvatae* load was measured via qRT-PCR using specific primers Ars16S (Supplementary Table S1). The relative quantity of *Arsenophonus* was calculated based on the 2^-ΔΔCT^ method [31]. The PCR protocol is described in more detail in Supplementary Methods.

### Histological and ultrastructural microscopy

GFP fluorescence of eggs, nymphs, and adults was observed using an Olympus SZX16 stereoscope (Olympus, Japan). For electron microscopy, ovaries were dissected rapidly in phosphate-buffered saline (PBS) and fixed in glutaraldehyde in PBS overnight, followed by dehydration in ethanol, embedding, and sectioning. Ultrathin sections were stained with uranyl acetate and lead citrate before being imaged using a Hitachi HT-7800 transmission electron microscope (Hitachi High-Tech Corporation, Japan) (see Supplementary Methods).

### Insecticide bioassay

Two different insecticide bioassays were performed according to target species. For *N. lugens*, the rice-seeding dip method was performed following our previous protocol [32], where third instar nymphs (Ca. *A. nilaparvatae-*GFP infected/uninfected) were introduced to rice seedlings soaked with different concentrations of nitenpyram, and the control was treated with 0.1% Triton X-100. LC_50_ was estimated by examining survival across a concentration series. For each nitenpyram concentration, 45 nymphs were divided into three replicates (15 individuals per replicate) and tested. Survival was scored 96 h after insecticide exposure. For *D. melanogaster*, the mixed feed method was adapted from that of Andrew et al. [33]. *D. melanogaster* pupae were injected with Ca. *A. nilaparvatae-*GFP injection and adults with fluorescence after eclosion selected for use in the insecticide bioassay. The adults of *D. melanogaster* were introduced into fly vials with different concentrations of nitenpyram diluted in the fly medium. For each nitenpyram concentration, 48 nymphs were divided into three replicates (16 individuals per replicate, 8 males, and 8 females) and tested. The survival rate was recorded at 96 h after insecticide exposure.

### RNA sequencing

For RNA sequencing, we selected *N. lugens* adults exhibiting green fluorescent signal six days after Ca. *A. nilaparvatae-*GFP injection (*N. lugens* individuals injected with water were used as controls). Every treatment had three replicates with 20 individuals per replicate, 10 males, and 10 females). Total RNA of third-instar larvae was extracted using the RNAiso Plus Kit (TaKaRa, Shiga, Japan) in accordance with the manufacturer’s instructions. Libraries were sequenced on the Illumina HiSeq platform by Shanghai Personal Biotechnology Co., Ltd. (Shanghai, China). RNA sequencing data analysis is described in more detail in Supplementary Methods.

### Quantification and statistical analysis

We analyzed the data using GraphPad Prism (version 7. 0; Graphpad Software, San Diego, CA, USA) to perform data plotting and statistical analyses. Specifically, we used Student’s *t*-test to compare the means between groups. The level of significance was set at *P* < .001 (^***^), *P* < .01 (^**^), *P* ≤ .05 (^*^), and *P* > .05 (ns). Bioassay data was analyzed using Probit analysis implemented on Polo Plus software (version 2.0) [34]. Initially, fit of data to probit model assumptions was tested for each treatment group using a likelihood approach; having established fit to model assumptions (not significant Chi squared report), LC_50_ was then calculated for each group with confidence intervals and statistical significance implied by nonoverlapping ranges; note this metric is conservative with respect to statistical significance [35]. Data plotting and statistical analyses were performed using GraphPad Prism (version 7.0) and R (4.3.2).

## Results

### Establishment of a primary Ca. *A. nilaparvatae* culture from *N. lugens* and genome sequencing

**Figure 1:**
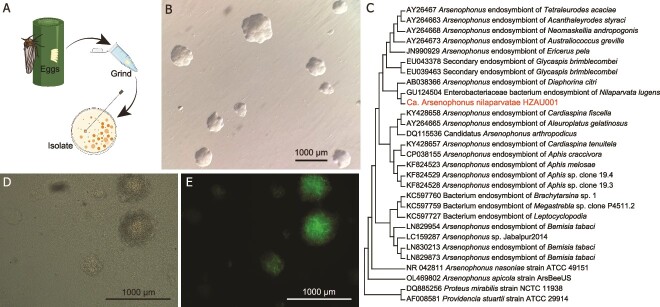
Establishment of a primary *Arsenophonus* culture from *N. lugens.* (A) Procedure for the establishment of a primary *Arsenophonus* culture from eggs of *N. lugens.* (B) Colony appearance of Ca. *A. nilaparvatae.* Ca. *A. nilaparvatae* colonies were small (1–2 mm), translucent, and of a wrinkled appearance on DNase agar. (C) Phylogenetic position of the cloned microbe based on 16S rRNA gene sequences confirms identity in genus *Arsenophonus*. (D and E) Colony appearance of Ca. *A. nilaparvatae*-GFP. (D) Light-microscopy images and (E) epifluorescence microscopy images of the Ca. *A. nilaparvatae* colonies.

The primary culture of Ca. *A. nilaparvatae* was derived from surface-sterilized eggs of *N. lugens* (Fig. 1A). Ca. *A. nilaparvatae* grew at 28°C in an aerobic environment. Growth was slow, forming small (1–2 mm), translucent colonies with a wrinkled appearance after 6 days on DNase agar (Fig. 1B). The appearance and slow growth of this microbe in culture closely parallels that of *A. nasoniae*, the type species for the genus [13]. Phylogenetic analysis of 16S rRNA gene sequences confirmed the isolated bacterium lay within the genus *Arsenophonus*, falling into the same taxonomic branch as the previously identified *Arsenophonus* endosymbionts of *N. lugens* (99.72% identity) and is most closely related to the *Arsenophonus* endosymbiont of *Diaphorina citri* (99.5% identity, Fig. 1C).

**Figure 2:**
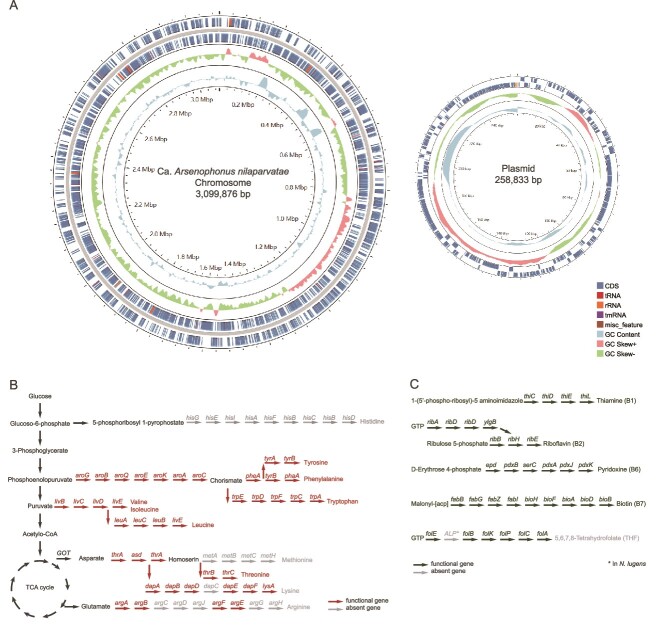
Genomic features and predicted biosynthetic capacity of Ca. *A. nilaparvatae.* (A) Ca. *A. nilaparvatae* main chromosome and plasmid. Biosynthesis pathways of amino acids (B) and B vitamins (C) are encoded in the genomes of Ca. *A. nilaparvatae.* Arrows represent individual enzymatic steps, with the abbreviated name of the gene shown above. Dark-colored arrows indicate the presence of the respective gene, and light-colored arrows the absence.

The cultured isolate enabled us to complete a closed assembly of the isolated strain HZAU001 using Nanopore and DNBSEQ platforms. The final assembly of Ca. *A. nilaparvatae* consists of a circular chromosome 3.10 Mb long, which is smaller than the type species *A. nasoniae* (3.87 Mb) and the other described member of the genus, *Arsenophonus apicola* (3.3 Mb) [36, 37]. The genome is considerably less complex than *A. nasoniae*, with the main Ca. *A. nilaparvatae* chromosome predicted to carry four intact prophage each of 40–50 kb and a single circular plasmid (259 kb) (Fig. 2A and Supplementary Table S2). Analysis of coding content indicates this plasmid is likely to be conjugative; the relative read depth of sequencing reads produce an estimate of 5.6 plasmid copies per main chromosome. The main chromosome has an average G + C content of 38.20%. A total of 3380 genes were predicted using Glimmer 3.02 (Supplementary Table S3).

The genome sequence has similar properties to the draft genome for the distinct isolate previously analyzed in terms of predicted metabolic capacity, including complete biosynthetic pathways for the essential amino acids like phenylalanine, tryptophan, valine, leucine, isoleucine, and threonine (Fig. 2B) [38]. In addition to the pathways for essential amino acid biosynthesis, Ca. *A. nilaparvatae* appears to be capable of synthesizing multiple cofactors, including riboflavin (vitamin B2), cobalamin (vitamin B12), and folate (vitamin B9) (Fig. 2C). The genome encodes capacity for both flagella and fimbriae.

The genome encoded type III and type VI secretion systems and a range of secreted effector molecules. Aside type III secreted effectors, there were a range of genes predicted to encode RTX (repeat in toxin) genes with sequence homology to serralysin or haemolysin, the former a Ca^2+^-dependent toxin produced by *Serratia*, alongside an ABC system predicted to transport RTX toxins across the bacterial membrane. In addition, there were six copies of a gene with sequence similarity and domains of the zona occludens toxin (*zot*) gene of *Vibrio cholerae*. Functional studies of zona occludens toxin from *V. cholerae* indicate this protein enables transfer of small molecules through the tight junctions of epithelia, and it is likely this function represents an important component of the symbiotic interaction [39]. There were also a few genes with sequence similarity to elements of the *Tc* toxin complex (Supplementary Table S4). In addition, the genome possessed a non-ribosomal peptide synthesis (NRPS) predicted to synthesize three small molecules including catechol, a precursor for enterobactin siderophore production, and phenethylamine (Supplementary Table S5). Overall, the microbial genome presented many features associated with the capacity of this microbe to interface with and alter host biology. These features are most commonly found in infectious pathogens and heritable symbionts that have recently transitioned from a pathogenic lifestyle.

### Transformation of Ca. *A. nilaparvatae* enables elucidation of somatic and vertical transmission processes in *N. lugens*.

Ca. *A. nilaparvatae* was transformed with plasmid pOM1-*gfp in vitro* and we named the resultant strain *A. nilaparvatae*-GFP [29, 40]. This clone exhibited the anticipated bright-green fluorescence when compared to the controls (Fig. 1D and E).

**Figure 3:**
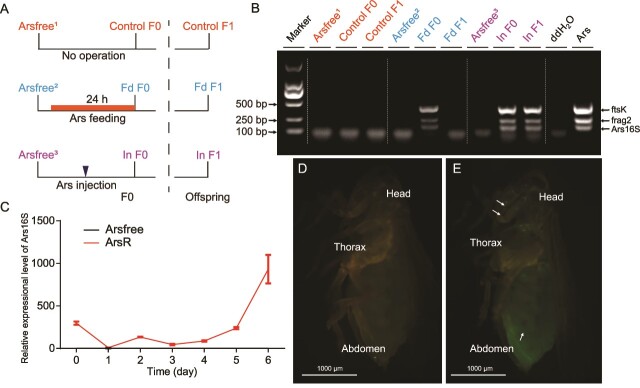
Somatic infection of Ca. *A. nilaparvatae* in *N. lugens* following feeding and injection routes*.* (A) Scheme of the experimental procedure for artificial infection method of Ca. *A. nilaparvatae*. Arsfree is *N. lugens* uninfected with Ca. *A. nilaparvatae.* “Fd” represents introduction through feeding and “In” represents introduction through injection. (B) PCR detection of Ca. *A. nilaparvatae* in different samples after artificial infection by method from (A). Three specific sequences (Supplementary Table S1) were used to determine presence of Ca. *A. nilaparvatae.* (C) Growth dynamics of Ca. *A. nilaparvatae* following injection. The relative expression level of Ars 16S rRNA was detected by qRT-PCR. “ArsR” represents *Arsenophonus*-reintroduce. Bars reflect the mean ± standard error of the mean (SEM). (D and E) Progression of Ca. *A. nilaparvatae-*GFP infection of *N. lugens* adult. Fluorescent microscopy images of Ca. *A. nilaparvatae* free *N. lugens* (D) and Ca. *A. nilaparvatae* injected *N. lugens* (E).

We developed an artificial infection method to reintroduce the symbiont to *N. lugens* insects. When Ca. *A. nilaparvatae*-GFP was dispersed in sterile water and fed directly to *N. lugens*, only a small titre of Ca. *A. nilaparvatae*-GFP could be detected in *N. lugens*, indicating passage from gut to inside the *N. lugens* body presented an impediment to establishment (Fig. 3A). Subsequently, we establish infection directly through microinjection of culture into the haemolymph. This method resulted in *in vivo* colonization (Fig. 3B). qRT-PCR showed that *A. nilaparvatae*-GFP was present at low titre in a focal infection at three days, and then proliferated about four days postinjection (Fig. 3C and Supplementary Video S1). Through observation, we observed that *A. nilaparvatae*-GFP cells established disseminated infections through the haemolymph (Fig. 3D and E).

**Figure 4:**
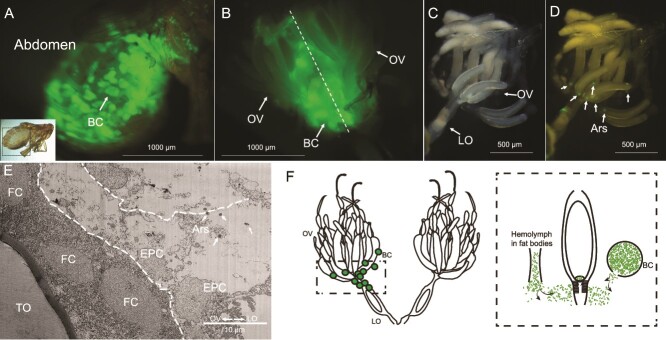
Vertical transmission process of Ca. *A. nilaparvatae* in *N. lugens.* (A) Side view of the abdomen of *N. lugens* female adults infected with Ca. *A. nilaparvatae-*GFP. (B) Anatomical photos of the abdomen of *N. lugens* female adults infected with Ca. *A. nilaparvatae-*GFP. (C and D) Localization of Ca. *A. nilaparvatae* in oocytes within the ovary. (C) Light microscopy and (D) epifluorescence images. (E) TEM images of the posterior region of the ovary, where the arrowheads depict Ca. *A. nilaparvatae*. (F) Schematic illustrations of vertical transmission process of Ca. *A. nilaparvatae* from fat body to ovary of *N. lugens.* BC, bacteriome; OV, ovary; LO, lateral oviduct; FC, follicle cell; TO, terminal oocyte; EPC, epithelial plug cell.

Vertical transmission of the symbiont was observed, with our transinfected mothers producing a mix of eggs infected and uninfected with the symbiont (Supplementary Fig. S1). We used the resultant Ca. *A. nilaparvatae*-GFP line to document the process of symbiosis development from an adult female to her progeny through vertical transmission, and thence from the infected zygote through to the adult insect. When observing female adult *N. lugens*, we observed Ca. *A. nilaparvatae*-GFP within the abdomen of its host (Fig. 4A). As previously shown by other groups working on this insect, bacteriocytes resemble fat body cells and are broadly distributed through the abdomen of *N. lugens* [41]. We further observed that the ovary of *N. lugens* is also covered with bacteriocytes (Fig. 4B). After surface cleaning these bacteriocytes, it was evident that the formed oocyte inside the ovary contained discrete foci of GFP fluorescence, indicating that Ca. *A. nilaparvatae*-GFP enter the ovaries during the formation of *N. lugens* oocytes (Fig. 4C and D). Transmission electron microscopy revealed that the follicular cells forming the epithelial plug are used by Ca. *A. nilaparvatae* to enter the terminal oocyte (Fig. 4E, Supplementary Figs S2 and 3), while the bacteriocytes around the ovary may act as a reservoir of the endosymbiont (Fig. 4F).

**Figure 5:**
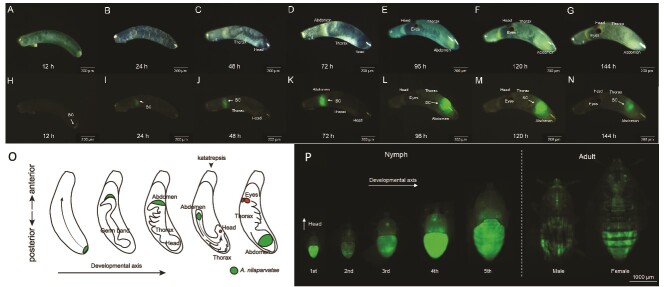
Morphological characteristics of embryos during the embryogenesis of *N. lugens* and dynamics of the bacteriocyte associated symbiont Ca. *A. nilaparvatae.* (A and H) Newly laid eggs. (B and I) Embryos 24 h after oviposition. (C and J) Embryos 48 h after oviposition. (D and K) Embryos 72 h after oviposition. (E and L) Embryos 96 h after oviposition. (F and M) Embryos 120 h after oviposition. (G and N) Embryos 144 h after oviposition. Panels A-G are light-microscopy images and H-N are fluorescent microscopy images in which green signals show the symbiotic bacterium. Arrowheads depict bacteriocytes. (O) Schematic illustrations of symbiont localization in the embryos. (P) Back view of nymphs of different ages and adults. BC, bacteriome.

We then tracked the fate of Ca. *A. nilaparvatae*-GFP in live developing embryos and through nymphal stages (Fig. 5), anchoring symbiont location to morphological landmarks of undissected eggs [42]. Twelve hour after egg laying (AEL), the *N. lugens* cells in the egg continued dividing (Fig. 5A). The symbionts remained at the posterior pole of the egg, mirroring their position in the oocyte (Fig. 5H). At 24 h AEL, the yolk was completely divided into small squares, and during this process, the symbionts were pushed towards the anterior pole of the egg (Fig. 5B and I and Supplementary Fig. S4). At 48 h AEL, the germ band becomes distinctly visible. The tail region of the embryo is localized near the anterior pole of the egg, and its head is point towards the posterior egg pole (Fig. 5C). At this point, the symbionts are located at the tail of the germband (Fig. 5G). Within this period, the embryo gradually became longer and wider, the head lobe, and thorax of the embryo began to form concomitantly, while segmentation began (Fig. 5D and K). At 96 h AEL, during the remodelling process (“embryonic flip”) called katatrepsis, the symbionts move form anterior to the posterior end of the egg by the embryo, a process that brings them into abdominal segments (Fig. 5E and L). The *N. lugens* eyes become larger and more darkly pigmented at 120 h AEL (Fig. 5F and M), and the abdominal segments sequentially form at 144 h AEL. At this point, Ca. *A. nilaparvatae*-GFP was, in some cases, observed more widely across the embryo, indicating dissemination of the symbiont from the previous highly restricted tropism (Fig. 5G and N). After egg hatching, Ca. *A. nilaparvatae*-GFP can be seen to establish a broadly disseminated infection in the abdomen of the *N. lugens* nymph (Fig. 5P and Supplementary Fig. S5). These results suggest a closely intertwined developmental relationship between Ca. *A. nilaparvatae* and *N. lugens*.

The frequency of symbionts in natural populations is very sensitive to the vertical transmission rate [43]. In our studies, we observed some eggs from infected mothers that were GFP-negative and inferred to be not infected alongside GFP-positive infected eggs (Supplementary Fig. S1), and other cases where infected embryos lost the infection during early development (Supplementary Fig. S3). Segregational loss of Ca. *A. nilaparvatae*-GFP is thus a composite of failure to enter the oocyte within the female, and failure of the symbiont to establish somatically during embryogenesis. We measured vertical transmission from three sets of infected females; vertical transmission efficiency was heterogeneous between the three sets (χ^2^ 2.d.f = 9.54; *P* = 0.008), with between 88.54%–97.50% of progeny acquiring infection (Supplementary Table S6).

### Ca. *A. nilaparvatae* can establish somatic, but not vertically transmitted, infection in heterologous hosts

**Figure 6:**
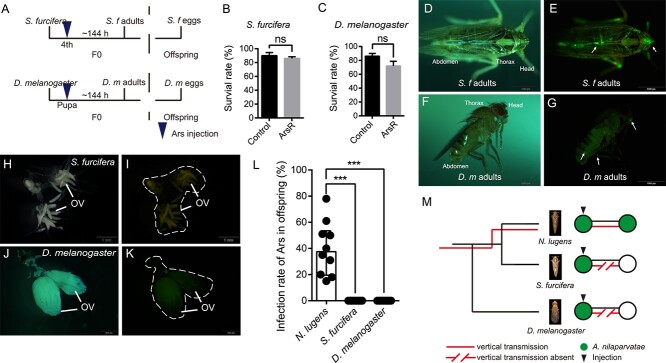
Absence of vertical transmission of Ca. *A. nilaparvatae* in novel host insect species*.* (A) Scheme of the experimental procedure for artificial infection method of Ca. *A. nilaparvatae* to novel host species (B and C) survival rate after Ca. *A. nilaparvatae* injection of *S. furcifera* (B) and *D. melanogaster* (C). “ArsR” represents *Arsenophonus*-reintroduce. (D and E) Images of *S. furcifera* adults infected with Ca. *A. nilaparvatae-*GFP. (F and G) Images of *D. melanogaster* adults infected with Ca. *A. nilaparvatae-*GFP. (H and I) Images of *S. furcifera* ovary infected with Ca. *A. nilaparvatae-*GFP. (G and K) Images of the *D. melanogaster* ovary infected with Ca. *A. nilaparvatae-*GFP. D, F, H, and J are light-microscopic images and E, G, I, and K are fluorescence microscopic images in which fluorescence signals show the symbiotic bacteria. (L) The infection rate of offspring of different species of insects after Ca. *A. nilaparvatae* infected (*n* = 500). (M) Schematic illustrations of the vertical transmission blockade of Ca. *A. nilaparvatae.* Statistical comparisons were based on *t*-tests, ns, no significant difference, and the level of significance for results was set at *P* < .001 (^***^).

We selected two host species in which to examine symbiont behaviour in heterologous hosts. The first of these, *Sogatella furcifera*, is a planthopper that also feeds on rice, occupying a similar position on the rice stem to *N. lugens*. This symbiont is likely to be naturally exposed to Ca. *A. nilaparvatae* through its shared ecological niche. Second, we examined *D. melanogaster* as a distantly related model insect species (Fig. 6A).

Injection of Ca. *A. nilaparvatae* into these two host species leads to infection in both insects without developing pathogenesis (Fig. 6B and C). Ca. *A. nilaparvatae* disseminates in the same manner through the whole body (Fig. 6D-G). Contrastingly, observation of the eggs and dissection of the ovaries revealed that Ca. *A. nilaparvatae* does not enter the embryo as in *N. lugens* (Fig. 6H-K) and endpoint PCR did not find evidence transmission of Ca. *A. nilaparvatae*-GFP to the offspring in the new hosts (Fig. 6L and Supplementary Fig. S7). These results indicate specificity of Ca. *A. nilaparvatae* towards *N. lugens* in terms of establishment of vertical transmission in this host species (Fig. 6M).

### Introduction of Ca. *A. nilaparvatae* to host insects induces insecticide susceptibility

Previous work had established that the presence of Ca. *A. nilaparvatae* in field collected *N. lugens* is associated with increased susceptibility of *N. lugens* to insecticide treatments. We used our transinfected lines that were established from clonal isolates of Ca. *A. nilaparvatae*-GFP to formally test the causality of this relationship. Having established our data conformed to the expected probit model across the concentration series within each treatment (Chi-squared values NS), we observed that *N. lugens* carrying Ca. *A. nilaparvatae* were more susceptible to nitenpyram than comparator *N. lugens* without the symbiont, with clearly non-overlapping confidence intervals for LC_50_ for *Arsenophonus* present vs absent insects (Table 1). In addition, we examined whether the insecticide-sensitivity phenotype was present in somatically infected heterologous host, *D. melanogaster*, compared to control *D. melanogaster* subject to a control injection protocol without Ca. *A. nilaparvatae*. *D. melanogaster* somatically infected with Ca. *A. nilaparvatae* were more susceptible to insecticide than sham injected controls. These data establish a causal basis to the previously observed association between Ca. *A. nilaparvatae* and insecticide sensitivity. Furthermore, there is evidence that the trait is expressed in heterologous host species.

**Table 1:** Susceptibility of *N. lugens* and *D. melanogaster* hosts to nitenpyram treatment without/with Ca. *A. nilaparvatae* infection.

Species	Ca. *A. nilaparvatae*	Number	Slope (SE)	LC_50_ (95%CI) (mg/L)	χ2 (df)	RR
*N. lugens*	−	270	2.23 (0.23)	19.55 (15.92–25.21)	4.87 (4)	−
	+	270	1.75 (0.19)	10.58 (8.37–13.30)	6.76 (4)	0.54
*D. melanogaster*	−	288	4.42 (0.50)	8.26 (7.26–9.42)	0.77 (3)	−
	+	288	3.41 (0.37)	5.71 (4.76–6.64)	4.84 (3)	0.69

Note: LC_50_, Lethal Concentration 50%; χ2 value measures compatibility with probit model assumptions within group; resistance ratio (RR) = LC_50_ of Ca. *A. nilaparvatae*^+^/LC50 of Ca. *A. nilaparvatae*^−^.

**Figure 7:**
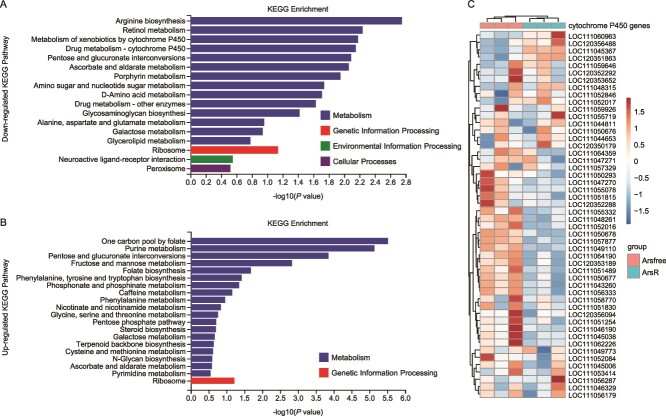
Transcriptome analysis of DEGs between Ca. *A. nilaparvatae* infected/uninfected *N. lugens*. (A) Enriched KEGG pathway analysis of *N. lugens* genes/pathways downregulated in the presence of Ca. *A. nilaparvatae* (B) enriched KEGG pathway analysis of *N. lugens* genes/pathways upregulated in the presence of Ca. *A. nilaparvatae*. A value of *P*_adj_ < .05 was used as the threshold for the significance of DEGs. (C) Heatmap of top 50 expression P450 genes of *N. lugens*. “ArsR” represents *Arsenophonus*-reintroduce.

Finally, we used RNAseq to test the causal basis of the transcriptional response of *N. lugens* to Ca. *A. nilaparvatae*. Previous work had observed P450 downregulation in *N. lugens* infected with S type Ca. *A. nilaparvatae* [22]. In our approach, we compared the transcriptional profile of adult *N. lugens* with Ca. *A. nilaparvataeA. nilaparvatae*-GFP introduced six days previously through injection vs *N. lugens* exposed to a sham injection in parallel. We observed that genes in the category “Metabolism of xenobiotics by P450” and “Drug metabolism- cytochrome P450” were downregulated in the presence of Ca. *A. nilaparvatae* (Fig. 7A and B). Analysis across individual genes within this category evidenced this effect was seen broadly across genes, though not in all genes within this category (Fig. 7C and Supplementary Table S7).

## Discussion

Symbioses between insects and heritable microorganisms are important to individual hosts, their ecology and evolution, and present opportunities for exploitation for novel and improved mechanisms of pest control and vector competence [44]. Within this field, the adaptation of microbes to endosymbiotic life—and the degraded genomes of many of these symbionts are a result of their restricted effective population size—present significant barriers to both functional research and exploitation [45]. This technical deficit is particularly notable in pest and vector species where symbionts remain a promising but underused tool. One particularly important trait in this respect is insecticide sensitivity, as insecticides represent the frontline measure to control pest insects on many crops worldwide and the extended use of insecticides has dramatic consequences in nontarget species [46]. Collectively, these factors make insecticide stewardship a pressing agenda.

We established Ca. *A. nilaparvatae*, the vertically transmitted symbiont of the brown planthopper, into cell-free culture. This symbiont—alongside the recently cultured Ca. Fukatsuia—clearly indicate the culturability of intracellular symbionts at the ca. 3 Mb genome size where culture-dependent approaches were previously considered difficult [16]. Our culture allowed completion of the closed genome (previously established as a draft genome from metagenomic approaches) and enabled bacterial transformation and the introduction of a plasmid encoding GFP expression. We utilized this strain to enable *in vivo* imaging of vertical and somatic development of infection in the brown planthopper host. Furthermore, bacterial cultures enabled an easy transfer to heterologous host species. Finally, reintroduction to the brown planthopper provided causal proof linking microbe presence to the trait of insecticide susceptibility at both the level of phenotype, and functionally in terms of downregulation of the *N. lugens* P450 detoxification system in the presence of Ca. *A. nilaparvatae*.

The means by which microbes establish vertical transmission varies greatly between symbioses [43]. For instance, *Wolbachia* enters oocytes through the somatic and germ line stem cell niches; *Spiroplasma* movement occurs through its attachment to vitellogenin, which is internalized later during oocyte development; *A. nasoniae* is acquired by the F1 larvae through feeding before developing foci of infection in the ovipositor [29, 47, 48]. Ca. *A. nilaparvatae* in *N. lugens* transfers from bacteriocytes to the posterior pole of the developing oocyte, forming a discrete focus of infection. This focus remains until late embryogenesis, at which point a disseminated somatic infection is established.

Whilst we generated somatic infections in two other insects—the closely related hemipteran pest *S. furcifera* and the model insect *D. melanogaster*, vertical transmission was not established in either case. Failure to move from somatic infection of a host into developing eggs is a known barrier for establishing heritable symbionts in novel host species [49, 50]. The causes of failure to vertically transmit are unclear in most cases, although inhibition by the gut microbiome is known to block *Wolbachia* movement from soma to germ line in *Anopheles* mosquitoes [51]. The capacity to vertically transmit in novel hosts presents a challenge for exploitation—whilst we can isolate, genetically manipulate, and return Ca. *A. nilaparvatae* to its native host and achieve onward vertical transmission, this complete transmission passage remains a challenge for heterologous host species where the microbe appears not to possess the genetic “key” for colonizing oocytes.

The major driver of research into the Ca. *A. nilaparvatae*—brown planthopper symbiosis is the economic importance of its host, and the previous observation that individuals infected with certain strains of Ca. *A. nilaparvatae* had increased sensitivity to insecticides compared to other field collected strains [22]. Our work—where pure clonal cultures of Ca. *A. nilaparvatae* were reintroduced to the host that then had higher insecticide susceptibility—completes causal proof of the role of Ca. *A. nilaparvatae* in this trait. This result has two consequences. First, where this strains of Ca. *A. nilaparvatae* is present, insecticide use will have higher efficiency and may be used with lower dose. Thus, simple testing of populations for this symbiont may direct principled use of insecticide. Reciprocally, field application of insecticide use may drive down frequency of the symbiont by disproportionately killing symbiont-infected planthoppers. Second, it may be possible to devise mechanisms to actively spread the symbiont through populations. If this spread can be achieved, we can essentially convert populations from insecticide resistant to insecticide sensitive, enabling improved productivity, reduced insecticide-associated costs, and reduced off target impacts. Furthermore, the culturability and transformability of symbionts such as Ca. *A. nilaparvatae* may be used to deliver targeted interventions, such as paratransgenic blocking of virus transmission from *N. lugens* to plants, for instance through delivery of cognate dsRNA from symbiont to host insect.

Symbionts represent an important new weapon in the fight against pests and vectors. Until now, application has been limited to the use of *Wolbachia* to control mosquito vector competence and population size [52, 53]. The development of these strategies across insects will necessitate a more detailed understanding of a broader range of symbionts, the phenotypic impact they have on their hosts, the ability to manipulate them and drive them into the population. Our work permits this analysis for the trait of insecticide susceptibility. Future work on the system should determine the drivers of symbiont spread alongside their functional biology. Examining the predicted coding capacity of the closed genome indicates the capacity for synthesis of essential amino acids and other cofactors that may contribute to *N. lugens* physiological and metabolic function, as well as a diverse armoury of genes allied to toxins. These toxins may be important in enabling symbiosis with the host, through impacting host physiological systems, or may function in protection of *N. lugens* against natural enemies, as observed in many other symbioses [54]. Research should also investigate infectious transmission potential, as recent work has established a broad range of insect symbionts that can transfer through plant hosts, including some *Arsenophonus* strains that transmit from hemipteran hosts to plants [55, 56]. Establishing if infectious transmission of Ca. *A. nilaparvatae* occurs at field-relevant rates is thus a pressing priority. Collectively, understanding these biological and ecological aspects of Ca. *A. nilaparvatae* will enable us to devise strategies to promote the spread of the symbiont, its intrinsic trait of enhanced susceptibility to insecticide, and the engineered traits to reduce its competence for viral transmission to rice.

## Supplementary Material

Supplementary_Materials_wrae194

Supplementary_Table_S7_wrae194

Video_S1_wrae194

## Data Availability

The genome sequence data of Ca. *A. nilaparvatae* has been deposited at GenBank (SAMN40534004) and the raw reads for *N. lugens* RNA-seq are available at the NCBI SRA (SAMN41895164 and SAMN41895163). They share the accession number PRJNA1089241.
