# Supplementary material for: Insecticide susceptibility in a planthopper pest increases following inoculation with cultured Arsenophonus
Source: ISME J. 2024 Oct 7;18(1):wrae194. doi: 10.1093/ismejo/wrae194 (PMC11491930; doi:10.1093/ismejo/wrae194)
Supplement: Supplementary_Materials_wrae194 [file supplementary_materials_wrae194.docx]

**Supplemental Materials for**

**Insecticide susceptibility in a planthopper pest increases following inoculation with cultured *Arsenophonus***

Tingwei Cai^1,2,5,6^, Pol Nadal-Jimenez^3,4^, Yuanyuan Gao^1,2,6^, Hiroshi Arai^4^, Chengyue Li^1,2,6^, Chunyan Su^1,2,6^, Kayla C King^5^, Shun He^6^, Jianhong Li^6^, Gregory D D Hurst^4^ and Hu Wan^1,2,6,^*

^1^ State Key Laboratory of Agricultural Microbiology, Huazhong Agricultural University, Wuhan 430070, China

^2^ Hubei Hongshan Laboratory, Wuhan 430070, China­

^3^ Department of Vector Biology, Liverpool School of Tropical Medicine, Liverpool, L3 5QA, United Kingdom

^4^ Institute of Infection, Veterinary and Ecological Sciences, University of Liverpool, Liverpool L69 7ZB, United Kingdom

^5^ Department of Microbiology & Immunology, University of British Columbia; Vancouver, British Columbia V6T 1Z3, Canada

^6^ Hubei Insect Resources Utilization and Sustainable Pest Management Key Laboratory, College of Plant Science and Technology, Huazhong Agricultural University, Wuhan 430070, China

*Corresponding authors: Hu Wan. State Key Laboratory of Agricultural Microbiology, Huazhong Agricultural University, Wuhan 430070, China. Email: [huwan@mail.hzau.edu.cn](mailto:huwan@mail.hzau.edu.cn)

**Supplemental Methods：**

**Insect Material**

An *Arsenophonus*-infected field population of *N. lugens* was originally collected from a rice paddy field in Xinyang City (31.58°N, 115.24°E), China, in 2017. *N. lugens* are maintained on rice seedlings at 28°C under 70%-80% relative humidity and a 16-hour light/8-hour dark photoperiod [1]. *Arsenophonus*-uninfected *N. lugens* (Ars^-^) in this population was allowed to expand. *S. furcifera* was originally collected from rice paddy fields in Jingzhou City (30.34°N, 112.50°E), China, in 2023 and maintained on the same conditions as *N. lugens. D. melanogaster* (strain Canton-S, CS) is maintained in the lab of the authors in a cornmeal-yeast agar medium (per 400 mL medium contains 350 mL water, 7.5% corn flour, 7.5% brown sugar, 0.5% yeast, and 0.5% agar) at 25°C with a photoperiod of 14 hours light/10-hours dark conditions (200 ± 10 eggs per 50 mL vial of medium in a 150 mL conical flask) [2]. Axenic flies were derived from dechorionated eggs as previously described [3]. Eggs deposited from conventional (Conv) mated females overnight were collected and rinsed 3 times in 75% ethanol and 0.6% sodium hypochlorite before inoculating onto autoclaved yeast-sucrose food in a biosafety cabinet.

**Symbiont Isolation, Morphology *in vitro* and Identification Through 16S rRNA Gene Sequence**

Eggs of *N. lugens* were peeled off from rice seedlings and surface sterilized by washing with 75% ethanol three times, before homogenization with sterile deionized water. The resultant liquid was spread without dilution on DNase agar (Qingdao Hope Bio-Technology Co. Ltd.). The medium was cultivated at 28°C under standard aerobic conditions. Subsequently, small bacterial colonies became evident after six days of growth.

This bacterial strain underwent five generations of purification before identification and the 16S rRNA gene sequence of colonies was obtained from the PCR product amplified using primers 27F/1492R (Supplementary Table S1). Polymerase chain reaction (PCR) with 20 μL reactions containing 10 μL of the Hieff™ PCR Master Mix contains Hieff™ Taq DNA Polymerase (Yeasen Biotechnology Co., Ltd) at the following thermal cycle: initial denaturation at 95°C for 3 min, followed by 35 cycles of 95°C for 30 s, 50°C for 30 s and 72°C for 1 min, followed by a final extension step at 72°C for 4 min. The product was cleaned and Sanger sequenced using the original primers (Tsingke Biotechnology Co. Ltd.). The strain was preserved at -80°C in 20% glycerol stocks and then grown routinely on brain heart infusion (BHI; Coolaber) medium.

**Symbiont Genome Sequencing, Assembly and Annotation**

*Ca.* A. nilaparvatae was grown in BHI media for five days. After this period, the culture was spun down in 50 mL centrifuge tubes at 20,000g for 10 min. The collected pellet of *Ca.* A. nilaparvatae was rapidly transferred to liquid nitrogen. The *Ca.* A. nilaparvatae strain HZAU001 genome DNA extraction was performed using magnetic beads following protocol No. BGI-PB-TQ-DNA-001 and sequenced using a Nanopore and DNBSEQ platform at the Beijing Genomics Institute (BGI, Shenzhen, China). For downstream analyses, the DNBSEQ and Nanopore reads were filtered using SOAPnuke (15.6) and porechop (0.2.4), respectively. For DNBSEQ data, we filtered out reads with a certain proportion of low quality (≤20) bases. For Nanopore data, reads with mean quality lower than 9 and shorter than 2000 bp were also removed. The cleaned Nanopore reads were assembled using Flye 2.9.3-b1797 with the nano-raw mode [4]. The assembled two closed circular contigs were polished with the DNBSEQ data using Pilon.1.24 for six times until no changes were recorded [5]. The resulting polished genome was annotated using the NCBI Prokaryotic Genome Annotation Pipeline (PGAP). Gene prediction was also performed on the *Ca.* A. nilaparvatae genome assembly by glimmer V3.02 with Hidden Markov models [6]. Prophage regions were predicted using PHASTEST web server (https://phastest.ca/submissions) and CRISPR identification using CRISPRFinder (https://crisprcas.i2bc.paris-saclay.fr/CrisprCasFinder/Index). Predicted NRPS were identified using SEMPI2.0 ([http://sempi.pharmazie.uni-freiburg.de/index](http://sempi.pharmazie.uni-freiburg.de/index)). The best hit using the Blast alignment tool was extracted for function annotation. Kyoto Encyclopedia of Genes and Genomes (KEGG), COG (Clusters of Orthologous Groups), NR (Non-Redundant Protein Database), InterPro (IPR), Swiss-Prot, and GO (Gene Ontology) database was used for general function annotation. Gene content was also manually examined for known virulence factors and systems.

**Genetic Manipulation of *Ca* A. nilaparvatae**

Fluorescent strains of *Ca.* A. nilaparvatae were obtained following the method as previously described [7]. Take a frozen strain of *Ca.* A. nilaparvatae for about 5 generations of activation and purification. Then an active culture of *Ca.* A. nilaparvatae was grown in BHI agar and then used to inoculate 50 mL of BHI. After six days period, the culture was spun down in 50 mL Greinier tubes at 20,000 g for 10 min. The pellet was re-suspended in 10% sterile glycerol and washed five times using 1 mL of sterile 10% glycerol in a regular Sigma 1–14 centrifuge at 16,160g for 1 min and re-suspended in 50 μL of the same solution. The pellet was then placed on ice for 10 min, and 200 ng of plasmid pOM1-*gfp* was added and gently mixed in the suspension. The mixture was then placed on ice for 10 additional minutes and electroporated in a 1 mm cuvette using 1 pulse at 2.6 kV in a micropulser electroporator (Bio-Rad, UK). Immediately after, 1 mL of BHI was added to the cuvette and the suspension was placed in a regular 5 mL sterile vial and allowed to recover at 30°C and 250 r.p.m. over 24 h. After this period the culture was pelleted and plated on BHI agar plates containing 50 μg mL^−1^ spectinomycin (Sigma). The plate was sealed to avoid desiccation and placed at 30°C for 6 days until tiny colonies appeared on the agar surface. GFP fluorescence was detected in an Olympus SZX16 stereoscope (Olympus, Japan).

**Artificial Infection of *N. lugens* with *Ca.* A. nilaparvatae**

Two main methods were used for artificial infection: feeding and injection. In brief, a bacterial suspension of *Ca.* A. nilaparvatae-GFP was prepared. To this end, *Ca.* A. nilaparvatae-GFP was grown in BHI to an OD_600_ = 0.2. Subsequently, one mL culture was spun down in 1.5 mL tubes at 12,000 g for 5 min. The supernatant was discarded and 100 μL sterile water added to create the inoculum. In the feeding group, 20 nymphs of *N. lugens* were released into a glass tube (2 cm × 15 cm) containing 100 μL of bacterial suspension, sandwiched between two membranes of stretched parafilm [8]. Samples were collected after 24 h feeding and the rest of nymphs of *N. lugens* were transferred to normal rice seedlings for incubation until the next generation was sampled. In the injection treatment, each *N. lugens* nymph was injected with 50 nL of bacterial suspension into the ventral abdomen and then placed on rice seedlings for rearing. Samples collection time points of injection treatment are the same as those for the feeding treatment (Fig. 3A).

**Specific Detection and Relative Quantification of *Ca.* A. nilaparvatae**

Total genomic DNA was extracted from the whole bodies of *N. lugens* using the FastDNA SPIN Kit (MP, Biomedicals, California, USA) for soil following the manufacturer's protocol. We employed three pairs of primers (ArsftsK, Ars16S and Arsfrag, Supplementary Table S1) for the specific detection of infection of *Ca.* A. nilaparvatae*.* The amplification system used the previously noted parameters at the following thermal cycle: initial denaturation at 95°C for 3 min, followed by 35 cycles of 95°C for 10 s, 55°C for 20 s and 72°C for 30 s, followed by a final extension step at 72°C for 4 min. The relative *Ca.* A. nilaparvatae load was measured via qRT-PCR using specific primers Ars16S (Supplementary Table S1). Subsequently, the DNA was used as the template for qRT-PCR with 20 μL reactions containing 10 μL of the Hieff® qPCR SYBR Green Master Mix (No Rox) (Yeasen Biotechnology Co., Ltd) and 100 nM of the primers. Housekeeping gene Nl18S was employed as the reference to normalize to the quantity of *N. lugens* DNA. PCR reactions were run in a BioRad CFX384 (Bio-Rad, CA, USA) at the following thermal cycle: initial denaturation at 95°C for 30 s, followed by 40 cycles of 95°C for 5 s and 60°C for 10 s. After the thermal cycles, a melt curve analysis was conducted from 55°C to 95°C. The relative quantity of *Arsenophonus* was calculated based on the 2^-ΔΔCT^ method [9].

**Histological and Ultrastructural Microscopy**

GFP fluorescence of eggs, nymphs, and adults was detected in an Olympus SZX16 stereoscope (Olympus, Japan). For electron microscopy, the ovaries were dissected rapidly in 0.1 M phosphate-buffered saline (PBS) (pH 7.2) and fixed in 2.5% glutaraldehyde in 0.1 M PBS (pH 7.2) at 4°C overnight. After rinsing with PBS three times, the samples were post-fixed with 1% osmium tetroxide (OsO4) in 0.1 M PBS for 2 h at 4°C. After washing with PBS three times, the samples were dehydrated in a graded ethanol series (30%, 50%, 70%, 80%, and 95% for 15 min; and 100% for 20 min three times). Finally, the samples were treated with pure acetone for 20 min; treated with a mixture of embedding agent and acetone (V/V = 1/1) for 1 h; treated with a mixture of embedding agent and acetone (V/V = 3/1) for 3 h; treated with pure embedding agent overnight; and the osmotically-treated samples were embedded, and heated at 70°C overnight to obtain the embedded samples. The samples were then sliced in an ultrathin microtome to obtain 70-90 nm sections, which were stained by a lead citrate solution and a 50% uranyl acetate ethanol solution for 5-10 min each, and then allowed to air dry before being observed in a transmission electron microscope, Hitachi HT-7800 (Hitachi High-Tech Corporation, Japan).

**Vertical transmission efficiency**

To assess the vertical transmission efficiency of *Ca.* A. nilaparvatae, adult female *N. lugens* exhibiting strong green fluorescence signal were selected and placed individually on rice seedlings in glass tubes for oviposition. After a 24-hour egg-laying period, the females were collected and their infection status was confirmed via PCR. Once the offspring hatched, the number of nymphs exhibiting green fluorescence was recorded, and PCR was performed on these individuals to further validate the presence of the *Ca.* A. nilaparvatae infection. Vertical transmission efficiency was calculated based on the proportion of fluorescent, PCR-positive offspring.

**RNA Sequencing**

For RNA sequencing, we selected *N. lugens* adults exhibiting green fluorescent signal six days after *Ca.* A. nilaparvatae*-*GFP injection (*N.lugens* individuals injected with water were used as controls). Every treatment had three replicates with 20 individuals per replicate, 10 males and 10 females). Total RNA of third-instar larvae was extracted using the RNAiso Plus Kit (TaKaRa, Shiga, Japan) in accordance with the instructions of the manufacturer. The sequencing libraries were sequenced on the Illumina HiSeq platform by Shanghai Personal Biotechnology Co., Ltd. (Shanghai, China). Clean reads were obtained by removing raw reads containing adaptors or poly-N sequences and those with a low quality (<Q20). Filtered Reads were aligned to the reference genome (ASM1435652v1) by HISAT2 (http://ccb.jhu.edu/software/hisat2/index.shtml). We used the HTSeq 2.0.3 statistical alignment to Read Count values on each gene, as the original expression of the gene. Expression level was normalized using FPKM. Transcripts were annotated based on the reference genome, and sequences were annotated to the Kyoto Encyclopedia of Genes and Genomes (KEGG) orthology (KO) database with the KEGG automatic annotation server. DESeq2 R package 1.16.1 was used to analyze the differential expression of the identified genes, and those with *p* < 0.05 and |log2(fold change) | ≥ 1 considered to be significantly differentially expressed. Gene ontology (GO) enrichment and KEGG pathway analysis (https://www.genome.jp/kegg/) were used to identify functional modules.

**Supplemental Figures：**


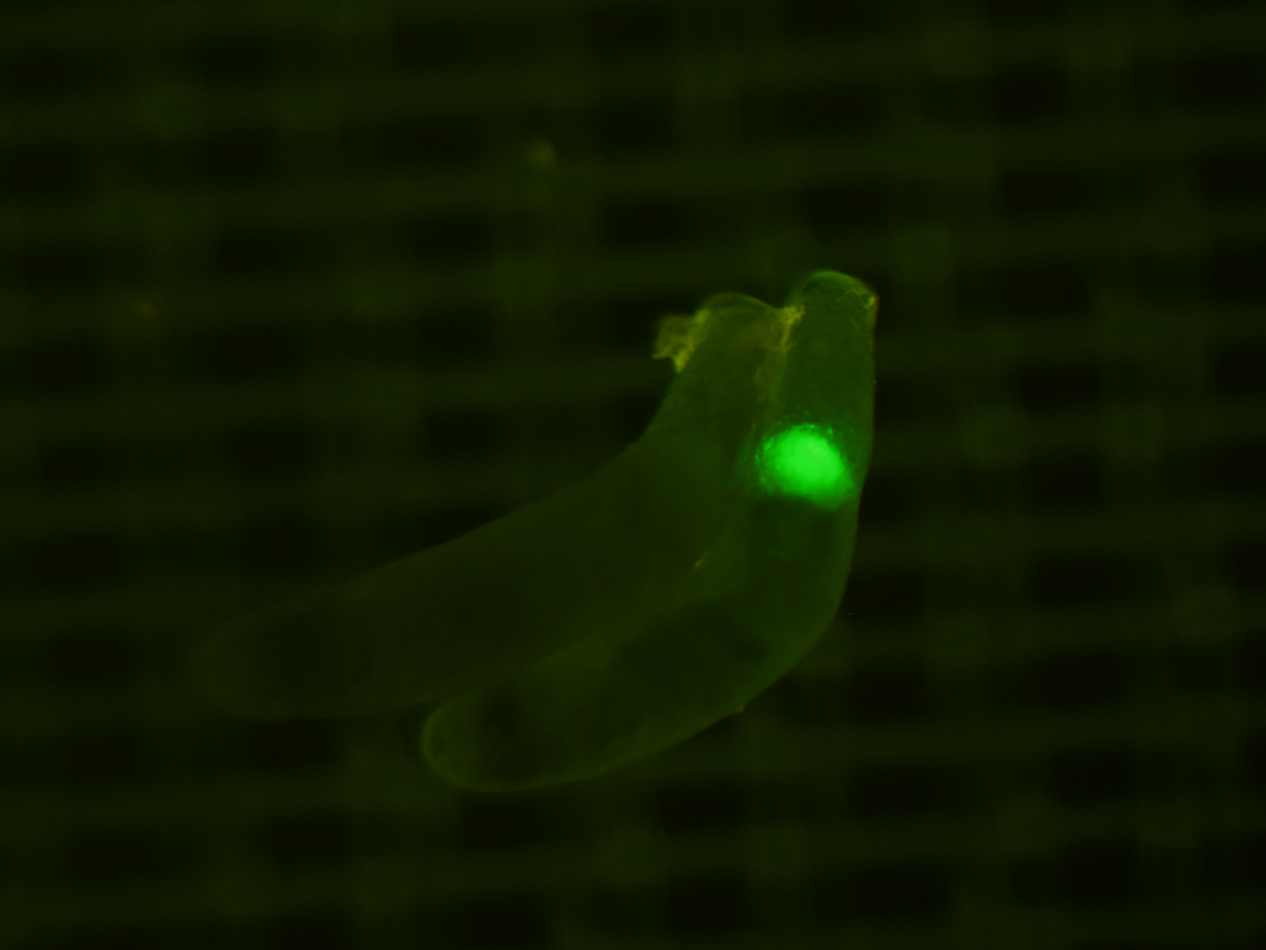


Fig. S1. The loss of vertical transmission of *Ca*. A. nilaparvatae. The photo shows different infection situations of *Ca*. A. nilaparvatae of eggs from the same female adult.


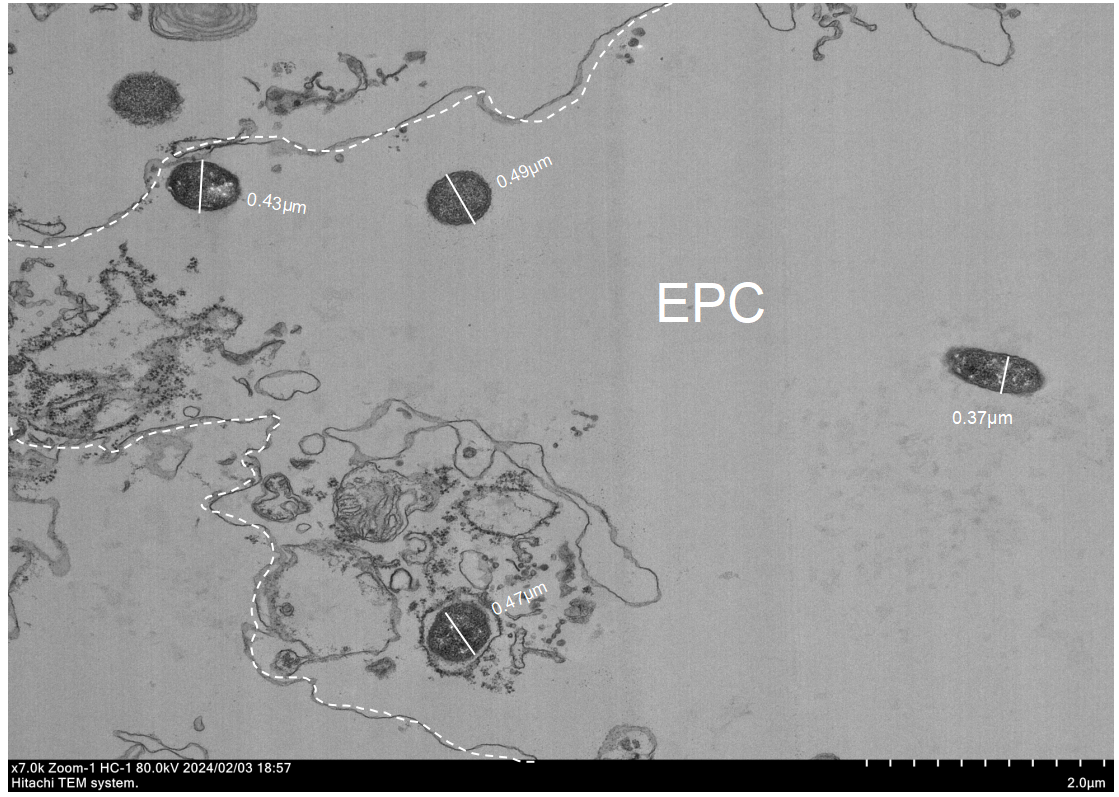


**Fig. S2 TEM images of the posterior region of the ovary.** where the The cross-sectional diameter of cell of *Ca.* A. nilaparvatae is represented by numbers.


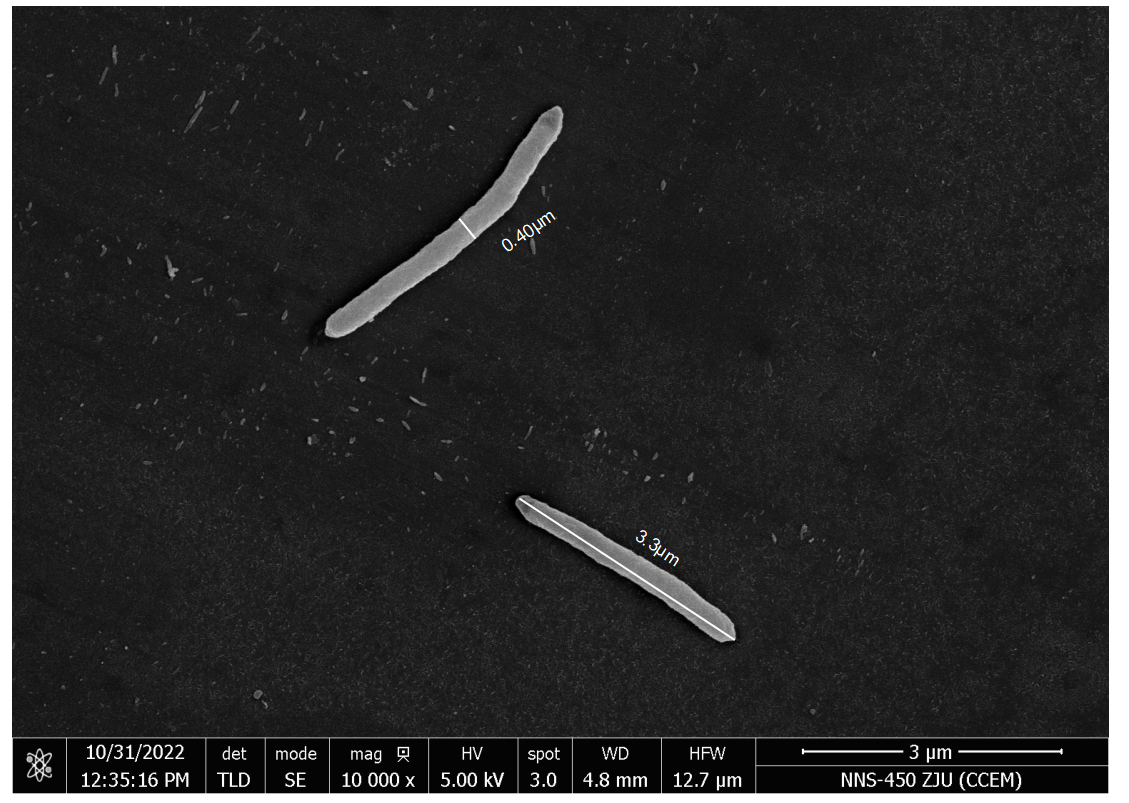


**Fig. S3**  **Scanning electron microscopy image of *Ca. A. nilaparvatae***

**
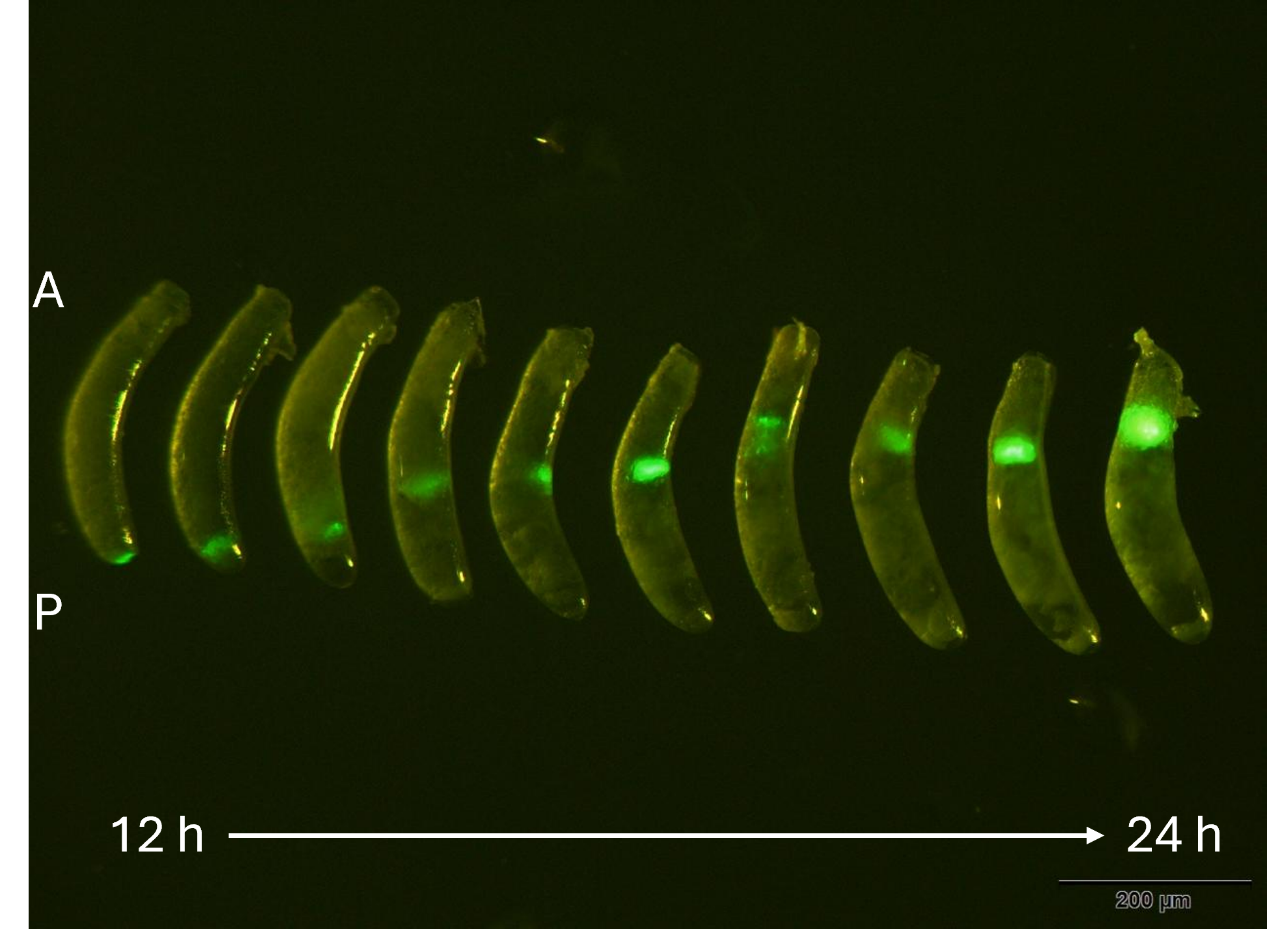
**

**Fig. S4**. **Localization of *Ca*. A. nilaparvatae in Eggs during 12- 24 h AEL.** The photo shows the continuous process of eggs at different developmental times with bacteriocytes pushed from the egg’s posterior (P) toward the anterio


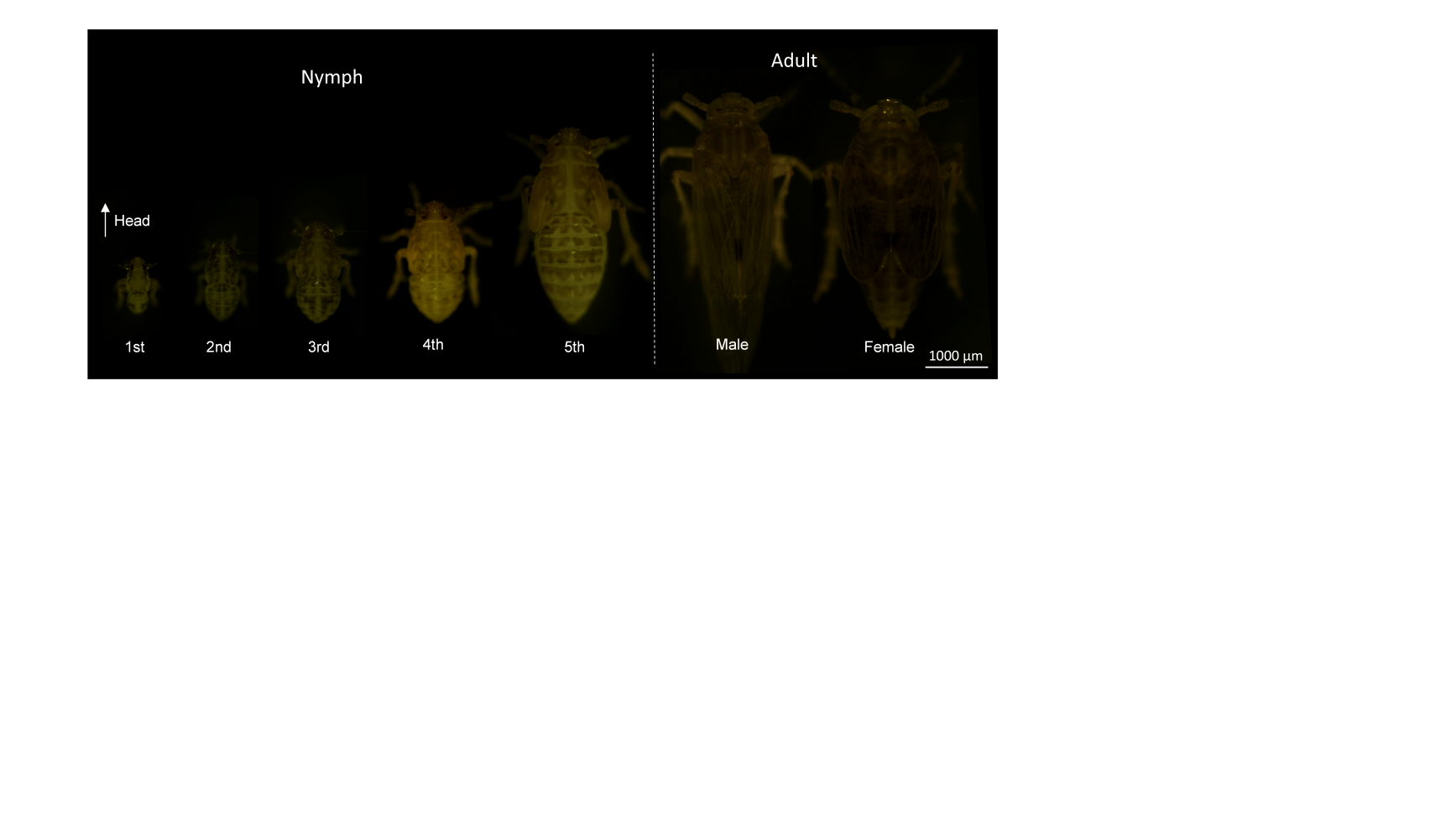


**Fig. S5**. **Morphological characteristics of the nymphs of *Nilaparvata lugens* and dynamics of the bacteriocyte associated symbiont *Ca*. Arsenophonus nilaparvatae.** Back view of nymphs of different ages and adults without *Ca.* A. nilaparvatae*-*GFP infection. Here is fluorescent microscopy images in which green signals show the symbiotic bacterium.


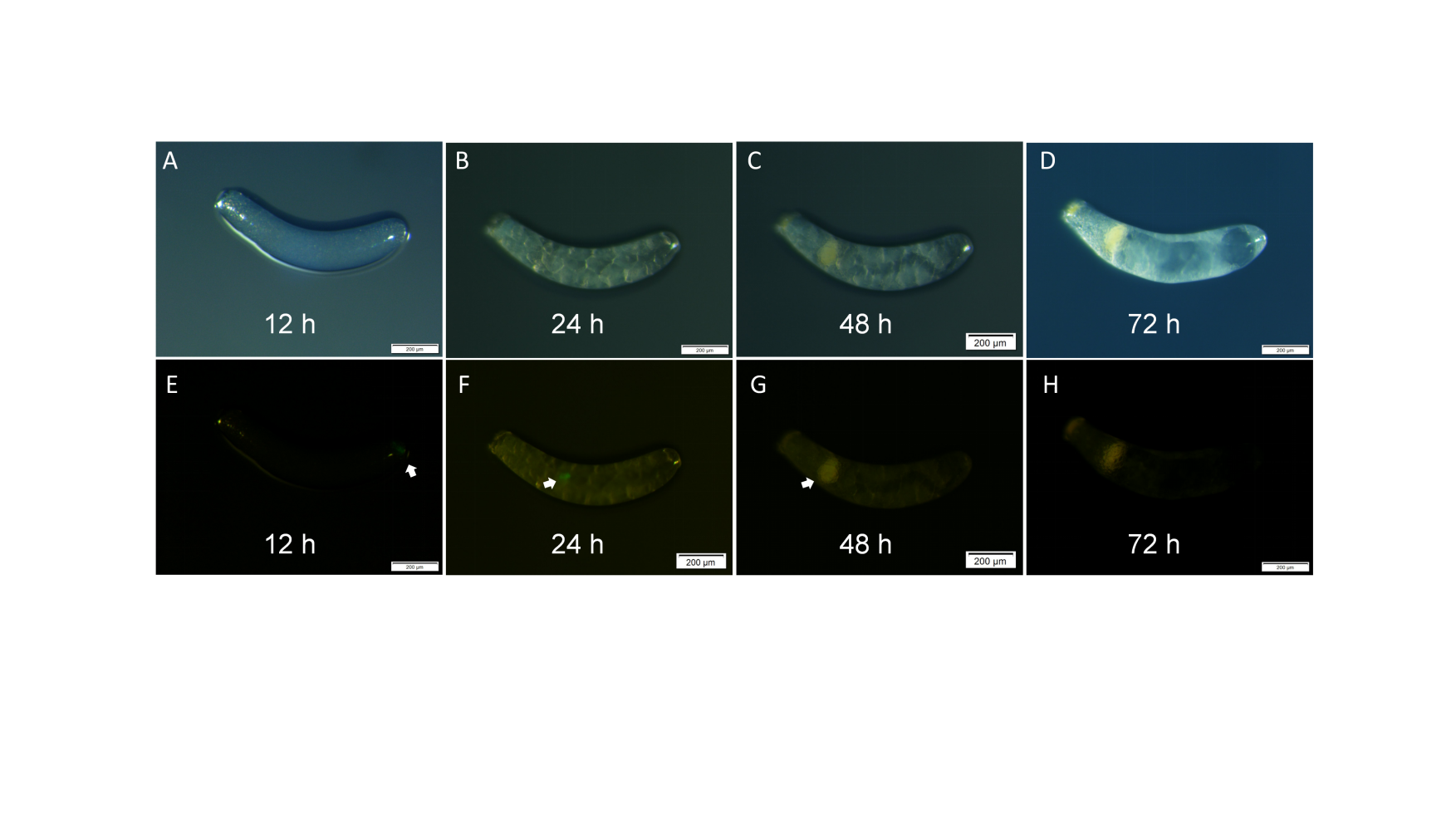


**Fig. S6. *Ca*. A. nilaparvatae infected embryos lost the infection during early development.** (A and E) Newly laid eggs. (B and F) Embryos 24 h after oviposition. (C and G) Embryos 48 h after oviposition. (D and H) Embryos 72 h after oviposition. A-D are light-microscopy images and E-H are fluorescent microscopy images in which green signals show the symbiotic bacterium. Arrowheads depict bacteriocytes. Fluorescence gradually disappears with the development of this egg.


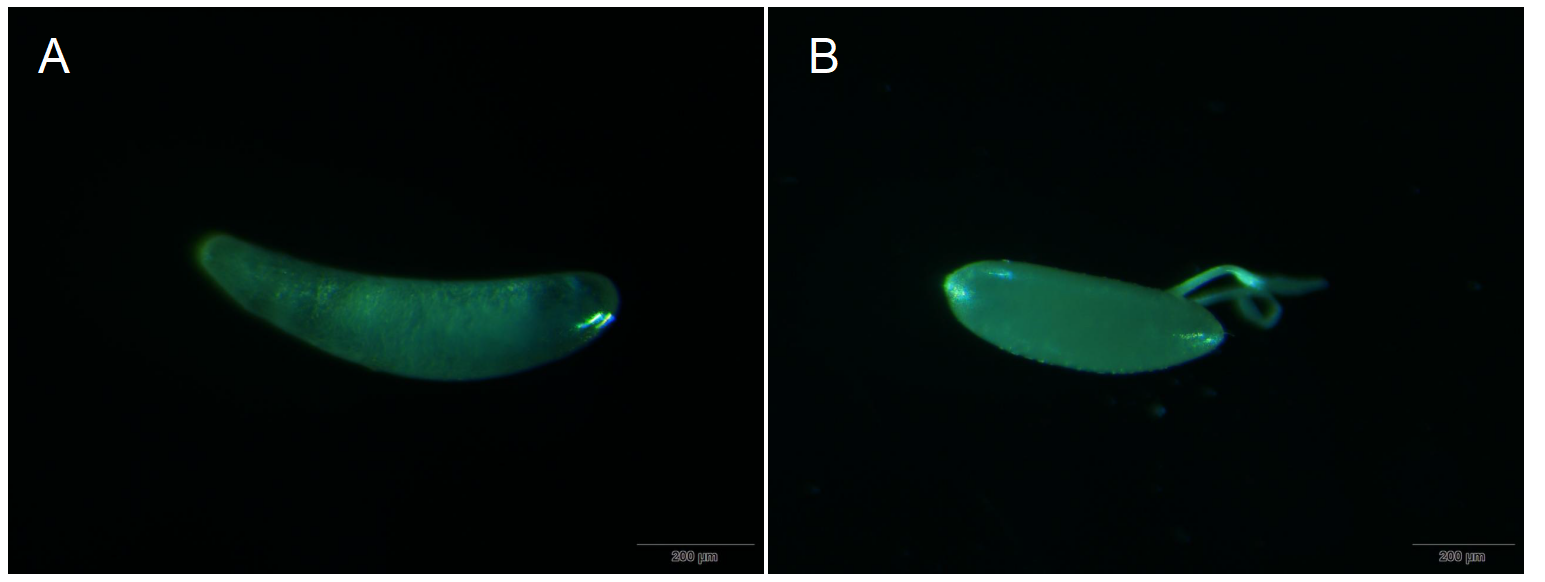


**Fig. S7. Absence of vertical transmission of *Ca.* Arsenophonus nilaparvatae in novel host insect species*.*** (A) Images of *S. furcifera* egg infected with *Ca.* A. nilaparvatae*-*GFP. (B) Images of *D. melanogaster* egg infected with *Ca.* A. nilaparvatae*-*GFP.

**Supplemental Tables：**

**Table S1 Primers table of this research**

| Primer name | Forward primer (5'-3') | Reverse primer (5'-3') |
| --- | --- | --- |
| 16SrRNA (27F/1492R) [10] | AGAGTTTGATCCTGGCTCAG | GGTTACCTTGTTACGACTT |
| ArsftsK [11] | GCCGATCTCATGATGACCG | CCATTACCACTCTCACCCTC |
| Ars16S [12] | TTCGGTCGGAACTCAAAGG | TCTGAGTTCCGCTTCCCATC |
| Arsfrag [13] | ATCGGTAACTTATTCGCCAGGT | CCCCACGACTATATTGCTGAAC |
| Nl18S^*^[14] | GTAACCCGCTGAACCTCCT | TCCGAAGACCTCACTAAATC |

* Reference genes for quantitative of bacterial symbionts in *N. lugens*.

| Sample Name | Sequence Type (#) | Sequence Topology | Sequence Number (#) | Total Length (bp) |
| --- | --- | --- | --- | --- |
| Ca. Arsenophonus nilaparvatae HZAU001 | Chromosome | circular | 1 | 3,099,876 |
|  | Plasmid | circular | 1 | 258,833 |

Table S2 Assembly Stat of Ca. Arsenophonus nilaparvatae HZAU001

Table S3 Genome Stat of *Ca*. Arsenophonus nilaparvatae HZAU001

| Parameter | Results |
| --- | --- |
| Total Sequence Length (bp): | 3,358,709 |
| Number of Sequences: | 2 |
| Longest Sequences (bp): | 3,099,876 |
| N50 (bp): | 3,099,876 |
| Gap Ratio (%): | 0 |
| GCcontent (%): | 38.2 |
| Number of CDSs: | 3,191 |
| Average Protein Length: | 290.3 |
| Coding Ratio (%): | 82.7 |
| Number of rRNAs: | 21 |
| Number of tRNAs: | 60 |
| CRISPR | 0 |
| Glimmer predicted genes | 3380 |
| KEGG | 1948 |
| phage | 4 |

**Table S4 Genome coded secreted effector molecules of *Ca*. Arsenophonus nilaparvatae**

| Name | NR:Subject_description |
| --- | --- |
| zona occludens toxin | zonular occludens toxin domain-containing protein [*Arsenophonus nasoniae*] |
|  | zonular occludens toxin domain-containing protein [*Arsenophonus nasoniae*] |
|  | zonular occludens toxin domain-containing protein [*Arsenophonus nasoniae*] |
|  | zonular occludens toxin domain-containing protein [*Arsenophonus nasoniae*] |
|  | zonular occludens toxin domain-containing protein [*Arsenophonus nasoniae*] |
|  | zonular occludens toxin domain-containing protein [*Arsenophonus nasoniae*] |
| type III secretion system | EscC/YscC/HrcC family type III secretion system outer membrane ring protein [*Candidatus* Arsenophonus nilaparvatae] |
|  | type III secretion system inner membrane ring subunit SctD [*Candidatus* Arsenophonus nilaparvatae] |
|  | EscF/YscF/HrpA family type III secretion system needle major subunit [*Candidatus* Arsenophonus nilaparvatae] |
|  | MULTISPECIES: type III secretion system needle filament subunit SctF [*Arsenophonus*] |
|  | MULTISPECIES: EscF/YscF/HrpA family type III secretion system needle major subunit [*Arsenophonus*] |
|  | MULTISPECIES: type III secretion inner membrane ring lipoprotein SctJ [*Arsenophonus*] |
|  | type III secretion inner membrane ring lipoprotein SctJ [*Candidatus* Arsenophonus nilaparvatae] |
|  | HrpE/YscL family type III secretion apparatus protein [*Candidatus* Arsenophonus nilaparvatae] |
|  | FliI/YscN family ATPase [*Candidatus* Arsenophonus nilaparvatae] |
|  | FliM/FliN family flagellar motor switch protein [*Candidatus* Arsenophonus nilaparvatae] |
|  | hypothetical protein [*Candidatus* Arsenophonus nilaparvatae] |
|  | FliM/FliN family flagellar motor switch protein [*Candidatus* Arsenophonus nilaparvatae] |
|  | MULTISPECIES: type III secretion system export apparatus subunit SctR [Arsenophonus] |
|  | type III secretion system export apparatus subunit SctS [*Arsenophonus* sp.] |
|  | type III secretion protein EscS,SsaS,YscS [*Arsenophonus nasoniae*] |
|  | type III secretion system export apparatus subunit SctT [*Candidatus* Arsenophonus nilaparvatae] |
|  | MULTISPECIES: type III secretion system export apparatus subunit SctT [Arsenophonus] |
|  | EscU/YscU/HrcU family type III secretion system export apparatus switch protein [*Candidatus* Arsenophonus nilaparvatae] |
|  | EscV/YscV/HrcV family type III secretion system export apparatus protein [*Candidatus* Arsenophonus nilaparvatae] |
|  | EscV/YscV/HrcV family type III secretion system export apparatus protein [*Candidatus* Arsenophonus nilaparvatae] |
|  | EscV/YscV/HrcV family type III secretion system export apparatus protein [*Candidatus* Arsenophonus nilaparvatae] |
|  | YscG family type III secretion protein [Arsenophonus sp.] |
|  | type III secretion system inner rod subunit SctI [*Candidatus* Arsenophonus nilaparvatae] |
|  | SctK family type III secretion system sorting platform protein [*Candidatus* Arsenophonus nilaparvatae] |
|  | type III secretion system inner rod subunit SctI [*Candidatus* Arsenophonus nilaparvatae] |
|  | type III secretion system inner rod subunit SctI [*Candidatus* Arsenophonus nilaparvatae] |
|  | PrgH/EprH family type III secretion apparatus protein [*Candidatus* Arsenophonus nilaparvatae] |
|  | type III secretion system outer membrane ring subunit SctC [*Candidatus* Arsenophonus nilaparvatae] |
|  | type III secretion system outer membrane ring subunit SctC [*Candidatus* Arsenophonus nilaparvatae] |
|  | type III secretion inner membrane ring lipoprotein SctJ [*Candidatus* Arsenophonus nilaparvatae] |
|  | type III secretion inner membrane ring lipoprotein SctJ [*Candidatus* Arsenophonus nilaparvatae] |
|  | type III secretion system ATPase SctN [*Candidatus* Arsenophonus nilaparvatae] |
|  | type III secretion system ATPase SctN [Arsenophonus sp.] |
|  | type III secretion system ATPase SctN [Arsenophonus sp.] |
|  | EscR/YscR/HrcR family type III secretion system export apparatus protein [*Candidatus* Arsenophonus nilaparvatae] |
|  | EscR/YscR/HrcR family type III secretion system export apparatus protein [*Candidatus* Arsenophonus nilaparvatae] |
|  | MULTISPECIES: EscS/YscS/HrcS family type III secretion system export apparatus protein [*Arsenophonus*] |
|  | MULTISPECIES: type III secretion system export apparatus subunit SctS [*Arsenophonus*] |
|  | type III secretion system export apparatus subunit SctT [*Candidatus* Arsenophonus nilaparvatae] |
|  | type III secretion system export apparatus subunit SctT [*Candidatus* Arsenophonus nilaparvatae] |
|  | EscU/YscU/HrcU family type III secretion system export apparatus switch protein [*Candidatus* Arsenophonus nilaparvatae] |
|  | EscU/YscU/HrcU family type III secretion system export apparatus switch protein [*Candidatus* Arsenophonus nilaparvatae] |
|  | type III secretion system gatekeeper subunit SctW [*Candidatus* Arsenophonus nilaparvatae] |
|  | type III secretion system gatekeeper subunit SctW [*Candidatus* Arsenophonus nilaparvatae] |
|  | hypothetical protein [*Candidatus* Arsenophonus nilaparvatae] |
|  | hypothetical protein [*Candidatus* Arsenophonus nilaparvatae] |
|  | hypothetical protein [*Candidatus* Arsenophonus nilaparvatae] |
| type III secreted effectors | MULTISPECIES: RNA chaperone ProQ [Arsenophonus] |
|  | serine/threonine-protein kinase [*Candidatus* Arsenophonus nilaparvatae] |
|  | hypothetical protein [*Candidatus* Arsenophonus nilaparvatae] |
|  | MULTISPECIES: secretion protein EspA [Arsenophonus] |
|  | secretion protein EspA [*Candidatus* Arsenophonus nilaparvatae] |
|  | type III secretion system translocon subunit SctE [*Candidatus* Arsenophonus nilaparvatae] |
|  | pathogenicity island effector protein [*Candidatus* Arsenophonus nilaparvatae] |
|  | hypothetical protein [*Candidatus* Arsenophonus nilaparvatae] |
|  | PipA/GogA/GtgA family type III secretion system effector [*Candidatus* Arsenophonus nilaparvatae] |
|  | PipA/GogA/GtgA family type III secretion system effector [*Candidatus* Arsenophonus nilaparvatae] |
| RTX toxin transport system | type I secretion system permease/ATPase [Arsenophonus sp.] |
|  | type I secretion system permease/ATPase [*Candidatus* Arsenophonus nilaparvatae] |
|  | HlyD family type I secretion periplasmic adaptor subunit [*Candidatus* Arsenophonus nilaparvatae] |
| serralysin | calcium-binding protein [*Candidatus* Arsenophonus nilaparvatae] |
|  | calcium-binding protein [*Candidatus* Arsenophonus nilaparvatae] |
|  | M10 family metallopeptidase C-terminal domain-containing protein [*Candidatus* Arsenophonus nilaparvatae] |
|  | M10 family metallopeptidase C-terminal domain-containing protein [*Arsenophonus* sp.] |
|  | calcium-binding protein [*Candidatus* Arsenophonus nilaparvatae] |
|  | M10 family metallopeptidase C-terminal domain-containing protein [Candidatus Arsenophonus nilaparvatae] |
|  | calcium-binding protein [*Arsenophonus nasoniae*] |
| hemolysin | MULTISPECIES: transcriptional regulator SlyA [Arsenophonus] |
|  | MULTISPECIES: CNNM family magnesium/cobalt transport protein CorC [*Arsenophonus*] |
| insecticidal toxin complex protein | RHS repeat-associated core domain-containing protein [Yersinia pseudotuberculosis] |
|  | RHS repeat-associated core domain-containing protein [Candidatus Arsenophonus nilaparvatae] |
|  | RHS repeat domain-containing protein [Candidatus Arsenophonus nilaparvatae] |
|  | RHS repeat domain-containing protein [Candidatus Arsenophonus nilaparvatae] |
|  | RHS repeat-associated core domain-containing protein [*Pseudomonas* fragi] |
|  | RHS repeat-associated core domain-containing protein [Candidatus Arsenophonus nilaparvatae] |
|  | RHS repeat-associated core domain-containing protein [Candidatus Arsenophonus nilaparvatae] |
|  | Tc toxin subunit A [Candidatus Arsenophonus nilaparvatae] |
|  | neuraminidase-like domain-containing protein [Candidatus Arsenophonus nilaparvatae] |
|  | toxin TcdB middle/C-terminal domain-containing protein [Candidatus Arsenophonus nilaparvatae] |
| other genes with sequence similarity to toxins. | anthrax toxin-like adenylyl cyclase domain-containing protein [Candidatus Arsenophonus nilaparvatae] |
|  | MULTISPECIES: type VI secretion system tube protein Hcp [*Arsenophonus*] |
|  | phospholipase [Candidatus Arsenophonus nilaparvatae] |
|  | enterotoxin A family protein [Candidatus Arsenophonus nilaparvatae] |
|  | Ail/Lom family outer membrane beta-barrel protein [Candidatus Arsenophonus nilaparvatae] |

| Cluster | Block | Type | DB screening | Cluster browser | Domains | Modify scaffold | Specificity | Smiles | Postsynthetic modifications | Figure |
| --- | --- | --- | --- | --- | --- | --- | --- | --- | --- | --- |
| 6 | 1 | nrps | Show | Open | Show | Scaffold Input | V * P | N[C@H](C(C)C)C(=O)N[C@H](C1CCCN1) | Sphingo:0 * Glyco:0 * Cl:0 * 6-Ring:0 * NO2:0 * SS:0 * 5-Ring:0 | NH2 O NH NH |
| 2 | 1 | nrps | Show | Open | Show | Scaffold Input | F | N[C@H](Cc1ccccc1) | None | H2N |
| 3 | 1 | nrps | Show | Open | Show | Scaffold Input | F | N[C@H](Cc1ccccc1) | Sphingo:0 * Glyco:0 * Cl:0 * 6-Ring:0 * NO2:0 * SS:0 * 5-Ring:0 | H2N |
| 1 | 1 | nrps | Show | Open | Show | Scaffold Input | F | N[C@H](Cc1ccccc1) | None | H2N |
| 5 | 1 | nrps | Show | Open | Show | Scaffold Input | dhb | Oc1c(O)c(ccc1) | None | HO HO |
| 7 | 1 | nrps | Show | Open | Show | Scaffold Input | F | N[C@H](Cc1ccccc1) | None | H2N |
| 4 | 1 | nrps | Show | Open | Show | Scaffold Input | F | N[C@H](Cc1ccccc1) | None | H2N |

**Table S5 Polyketides (type I) and nonribosomal peptides prediction and identification**

**Table S6 Vertical transmission (VT) efficiency**

| Number | F0 Total | F0 infected | F0-infected rate (%) | F1 Total | F1-infected | P (F1 infected) | 95% CI |
| --- | --- | --- | --- | --- | --- | --- | --- |
| 1 | 22 | 22 | 100 | 109 | 104 | 0.954 | 0.896 - 0.984 |
| 2 | 50 | 50 | 100 | 160 | 156 | 0.975 | 0.937 - 0.993 |
| 3 | 69 | 69 | 100 | 96 | 85 | 0.885 | 0.804 - 0.942 |

Note: VT efficiency = F1-infected rate/F0-infected rate*100. 95% CI, 95% confidence interval

**References**

1. Zhang Y et al. Decline in symbiont-dependent host detoxification metabolism contributes to increased insecticide susceptibility of insects under high temperature. *ISME Journal* 2021; **15**: 3693–3703. <https://doi.org/10.1038/s41396-021-01046-1>
2. Yamada R, Floate KD, Riegler M, O’Neill SL. Male development time influences the strength of *Wolbachia*-induced cytoplasmic incompatibility expression in *Drosophila melanogaster*. *Genetics* 2007. <https://doi.org/10.1534/genetics.106.068486>
3. Lee KA et al. Bacterial-derived uracil as a modulator of mucosal immunity and gut-microbe homeostasis in drosophila. *Cell* 2013; **153**: 797–811. <https://doi.org/10.1016/j.cell.2013.04.009>
4. Kolmogorov M, Yuan J, Lin Y, Pevzner PA. Assembly of long, error-prone reads using repeat graphs. *Nat Biotechnol* 2019; **37**: 540–546. <https://doi.org/10.1038/s41587-019-0072-8>
5. Walker BJ et al. Pilon: An integrated tool for comprehensive microbial variant detection and genome assembly improvement. *PLoS One* 2014; **9**. <https://doi.org/10.1371/journal.pone.0112963>
6. Salzberg SL, Delcher AL, Kasif S, White O. Microbial gene identification using interpolated Markov models. *Nucleic Acids Research* 1998. <https://doi.org/10.1093/nar/26.2.544>
7. Nadal-Jimenez P et al. Genetic manipulation allows in vivo tracking of the life cycle of the son-killer symbiont, *Arsenophonus nasoniae*, and reveals patterns of host invasion, tropism and pathology. *Environ Microbiol* 2019; **21**: 3172–3182. <https://doi.org/10.1111/1462-2920.14724>
8. Tang T et al. Antibiotics increased host insecticide susceptibility via collapsed bacterial symbionts reducing detoxification metabolism in the brown planthopper, *Nilaparvata lugens*. *J Pest Sci (2004)* 2020. <https://doi.org/10.1007/s10340-020-01294-8>
9. Livak KJ, Schmittgen TD. Analysis of relative gene expression data using real-time quantitative PCR and the 2^-ΔΔCT^ method. *Methods* 2001. <https://doi.org/10.1006/meth.2001.1262>
10. Srinivasan R *et al.*, Use of 16S rRNA Gene for Identification of a Broad Range of Clinically Relevant Bacterial Pathogens. *PLoS One* 2015; **10**: e0117617. <https://doi.org/10.1371/journal.pone.0117617>
11. Guo H et al. Interaction of *Arsenophonus* with *Wolbachia* in *Nilaparvata lugens*. *BMC Ecol Evol* 2021**; 21**: 31. <https://doi.org/10.1186/s12862-021-01766-0>
12. Mouton L *et al.*, Evidence of diversity and recombination in *Arsenophonus* symbionts of the *Bemisia tabacispecies* complex. *BMC Microbiol* 2012: **12**: S10. <https://doi.org/10.1186/1471-2180-12-S1-S10>
13. Pang R*et al.*, A distinct strain of *Arsenophonus* symbiont decreases insecticide resistance in its insect host. *PLoS Genet* 2018; **14**: e1007725. <https://doi.org/10.1371/journal.pgen.1007725>
14. Xi Y *et al.*, Chitinase-like gene family in the brown planthopper, *Nilaparvata lugens*. *Insect Mol Biol* 2015; **24**: 29–40. <https://doi.org/10.1111/imb.12133>
